# The Interplay between Cardiovascular Disease, Exercise, and the Gut Microbiome

**DOI:** 10.31083/j.rcm2311365

**Published:** 2022-10-27

**Authors:** Candace R. Longoria, John J. Guers, Sara C. Campbell

**Affiliations:** ^1^Department of Kinesiology and Health, Rutgers University, New Brunswick, NJ 08901, USA; ^2^Department of Biology, Behavioral Neuroscience and Health Science, Rider University, Lawrenceville, NJ 08646, USA

**Keywords:** gut microbiota, inflammation, endothelial function, prebiotics, probiotics, metabolites, trimethylamine N-oxide (TMAO)

## Abstract

Cardiovascular disease (CVD) is the leading cause of death worldwide, with physical inactivity being a known contributor to the global rates of CVD incidence. The gut microbiota has been associated with many diseases including CVD and other comorbidities such at type 2 diabetes and obesity. Researchers have begun to examine the gut microbiome as a predictor of early disease states by detecting disruptions, or dysbiosis, in the microbiota. Evidence is lacking to investigate the potential link between the gut microbiota, exercise, and CVD risk and development. Research supports that diets with whole food have reduced instances of CVD and associated diseases, increased abundances of beneficial gut bacteria, and altered gut-derived metabolite production. Further, exercise and lifestyle changes to increase physical activity demonstrate improved health outcomes related to CVD risk and comorbidities and gut microbial diversity. It is difficult to study an outcome such as CVD when including multiple factors; however, it is evident that exercise, lifestyle, and the gut microbiota contribute to improved health in their own ways. This review will highlight current research findings and what potential treatments of CVD may be generated by manipulation of the gut microbiota and/or exercise.

## 1. Introduction

Cardiovascular disease (CVD) is the leading cause of death and disability worldwide [1]. CVD is a disease of the heart and blood vessels which includes at least one of the following conditions: atherosclerosis, heart attack, stroke, heart failure, hypertension, and various other physical complications. The American Heart Association (AHA) classifies risk factors for CVD as either modifiable or nonmodifiable. Modifiable risks include tobacco use, alcohol consumption, high blood cholesterol and pressure, excessive body fat, diabetes, physical inactivity, and diet. Nonmodifiable risk factors include age, gender, and heritable issues. Physical inactivity is a known contributor to the global rates of CVD [2]. The United States “Physical Activity Guidelines for Americans” recommends adults engage in 150–300 minutes of moderate or 75 minutes of vigorous cardiovascular activity each week [2].

Exercise is known to alter gut microbiota diversity and short chain fatty acid (SCFA)-producing bacteria, particularly those that metabolize dietary fiber into butyrate [3, 4]. Butyrate has several positive effects on a range of metabolic and cardiovascular pathologies [5]. The gut microbiota has been associated with many diseases including CVD and other comorbidities such at type 2 diabetes (T2D) and obesity [5, 6, 7, 8]. In addition, the gut microbiota has been hypothesized to play a role in blood pressure regulation [9]. It is well established that hypertension is responsible for the onset of several chronic diseases. Furthermore, the gut microbiome is thought to be at least partially responsible for exercise-mediated protection from myocardial infarction in mice [10], while sedentary behavior in rodents tends to have a contrasting effect [9, 10]. Lastly, both supplementation with lactobacillus in rats [11] and fecal matter transplantation has been shown to improve outcomes of myocardial infarction and other cardiovascular pathologies, thus providing additional therapeutic opportunities [12].

Researchers have begun to examine the gut microbiome as a predictor of early disease states by detecting disruptions, or dysbiosis, in microbiota homeostasis [13, 14, 15]. In addition, diversity within the gut microbiota has long been touted as an indicator of overall health [15], however, others have indicated that interaction and activity of intestinal flora, not necessarily diversity, are major drivers of host health [16, 17].

Despite the connections between the gut microbiota and various chronic diseases [15], there is a lack of research investigating the potential link between the gut microbiota to CVD and less on how exercise can influence this potential relationship. CVD and its’ associated comorbid diseases (metabolic syndrome, T2D, and obesity) have been extensively reviewed [5, 6, 7, 8] and therefore will not be discussed in detail here. This review will highlight current research findings and what potential treatments of CVD may be generated by manipulation of the gut microbiota and/or exercise.

## 2. CVD may Present as at Least One of the Conditions Already Listed. Here, We Divide These Diseases into Two Categories: Diseases of the Vasculature and Diseases of the Heart. Within Each Section We will Discuss Disease Symptoms, Associated Changes Seen in the Gut Microbiota, and How Exercise may Impact Disease Risk and Development. 

### 2.1 Vascular Disease

#### 2.1.1 Endothelial Dysfunction and Arterial Stiffness

The endothelium is a thin, single layer of epithelial cells located between the blood vessel wall and circulating blood [18]. The action of blood flowing (sheer) over the thin endothelium can elicit responses from endothelial cells such as the release of nitric oxide (NO) and other signaling molecules. Endothelial dysfunction is described as both a loss of endothelium-dependent dilation and a deviation from normal functions which alter molecular signaling mechanisms, thereby decreasing the bioavailability of NO [18]. Over time, a decrease in NO bioavailability can lead to increased arterial stiffness and hypertension [19].

Aging is a natural contributor to vascular damage, endothelial dysfunction, and arterial stiffness [20]. Old mice (20–24 weeks) have lower endothelial dependent dilation (EDD), increased aortic wall thickness, and arterial stiffness in relation to young mice. Importantly, treatment of older mice with broad spectrum antibiotics attenuated losses in EDD and arterial stiffness [21]. *Ex vivo* data demonstrated this reduction in stiffness following antibiotic treatment may be due to reductions in aortic and carotid oxidative stress [21].

Trimethylamine is a gas which, when oxygenated, forms Trimethylamine N-oxide (TMAO) [22, 23]. Trimethylamine is a downstream product of L-carnitine. L-carnitine is found in abundance in dairy products, red meat, and fish. Collectively, TMAO production is the result of the gut microbiome metabolizing choline, betaine, and carnitine [23]. TMAO is commonly found in marine microbiota and is a substrate used for anaerobic metabolism in a number of bacteria [24]. Studies in humans have pointed to the fact that TMAO production is dependent on intestinal microbiota. This was demonstrated after plasma TMAO (pTMAO) levels were attenuated following the administration of broad-spectrum antibiotics [25].

There is a relationship between levels of TMAO and CVD. Human studies have shown that elevated levels are associated with the onset of atherosclerosis [26]. Mice fed a TMAO-supplemented diet for 6 months displayed decreased carotid artery EDD, which was induced by increases in oxidative stress; EDD was restored with the addition of the superoxide scavenging antioxidant, superoxide dismutase. Furthermore, *ex vivo* incubation of young mouse carotid arteries with various quantities of TMAO demonstrated that there is a dose-dependent response between TMAO levels and reductions in EDD [26].

Along with age-related increases in pTMAO concentrations, older mice exhibit a distinct clustering of the gut microbiota when observing development of arterial stiffness and endothelial dysfunction. Specifically, older mice are associated with an abundance of *Desulfovibrio*, *Akkermansia*, and *Bacteroides* and a decreased abundance of *Turicibacter*, *Clostridiaceae*, and *Parabacteroides* [21]. Following broad-spectrum antibiotic treatment, the age-related elevations in pTMAO were attenuated [21], supporting an influential role of the gut microbiome in altering pTMAO levels.

Evidence is limited in the relationship between endothelial dysfunction, arterial stiffness, and the gut microbiome. Further work targeting alterations in the gut microbiome and its associated metabolites will elucidate the interactions between disease development, progression, and severity.

#### 2.1.2 Hypertension

As per the American Heart Association (AHA), hypertension refers to excessive pressure placed on the walls of vasculature via circulating blood. Chronically elevated blood pressure places undue stress on the heart and blood vessels and over time can lead to pathological remodeling throughout the cardiovascular system. Ultimately, this can lead to conditions such as atherosclerosis and left ventricular hypertrophy. Pre-hypertensive and hypertensive rats [27, 28] and humans [15, 27, 29] have a less diverse gut microbiota relative to their respective normotensive counterparts. Using enterotype grouping, individuals with pre-hypertension and hypertension possessed a more *Prevotella*-dominant enterotype, whereas healthy controls possessed a more *Bacteroides*-dominant enterotype [15]. Evidence shows these enterotypes are diet-driven, with *Bacteroides *associated with animal protein, amino acids, and saturated fat and *Prevotella *associated with carbohydrates and simple sugars [30]. However, carbohydrate type and food quality, not necessarily a macronutrient group as a whole, may contribute to hypertension development [31]. Specifically, a metagenomic study of the gut microbiota of healthy elderly individuals identified the genus *Faecalicatena *to be cardioprotective against atherosclerosis and *Libanicoccus *to promote atherosclerosis [32]. Yan and colleagues [29] found hypertensive patients had increased abundances of *Klebsiella*, *Clostridium*, *Streptococcus*, *Parabacteroides, Eggerthella*, and *Salmonella* and decreased abundances of *Faecalibacterium *(*Faecalibacterium prausnitzii, (F. prausnitzii)*), *Roseburia*, and *Synergistetes. *Increased abundance of *Klebsiella *and decreased abundance of *Roseburia* are consistent with hypertensive individuals [15, 29]. *Klebsiella *has become well-known for its ability to quickly mutate, develop drug resistance, and act as an opportunistic pathogen that can cause infections [33]. *Roseburia *can produce lactate and utilize acetate to produce butyrate; it has also been found to grow best in medium containing inulins and various other fructans [34], which are known prebiotics [35].

In hypertensive elderly patients, *Lactobacillales*, *Blautia*, *Ruminococcus*, and *Escherichia coli* (*E. coli*) were negatively correlated with aerobic capacity [36]. Individuals with a ${\mathrm{VO}}_{2}$ peak greater than 20 mL/kg/min, showed enrichment of *Alcaligenaceae*, *Ruminococcaceae*, and *Faecalibacterium* compared to less trained individuals [36]. *Faecalibacterium *was consistently found have decreased abundances in hypertensive elderly patients and untrained individuals [36]. Interestingly *F. prausnitzii, *the only known species of the genus *Faecalibacterium*, is a known butyrate producer associated with anti-inflammatory activity [37]. These findings are consistent with our work showing that exercise training increases the abundance of *F. prausnitzii *in male mice [4], however more work associating specific microbes with human hypertensive patients needs to be conducted.

The spontaneous hypertensive rat (SHR) is a well-established animal model [28, 38], in which rats begin developing hypertension between 6–7 weeks old [39]. SHRs possesses a higher baseline abundance of the bacteria *Streptococcus*, *Parabacteroides*, and *Turicibacter* and a lower abundance of the bacteria *Coprococcus*, *Blautia, Allobaculum*, *Bifidobacterium*, and *Pseudobutyrivibrio *compared to control Wistar Kyoto (WKY) rats [27]. On the contrary, in a study by Li and colleagues [38], SHR vehicles possessed a decreased abundance of *Turicibacter* and *Romboustia *and an increase in *Helicobacteraceae *compared to WKY vehicle rats. These differences may be attributed to variations in gut microbiota analysis or housing conditions.

Following a 12-week, moderate intensity exercise protocol, the systolic blood pressure (SBP) of SHRs was significantly decreased; this decrease in SBP was maintained 4 weeks after exercise cessation [28]. Further, SHRs that were exercised had a significant increase in the abundance of *Turicibacter* and decreased abundance of *Helicobacteraceae * [38]. These findings in the SHR model demonstrate that significant decreases in SBP can occur as quickly as four weeks following moderate-intensity cardiovascular training and can decrease abundances of known pathogenic microbes.

### 2.2 Heart Disease

#### Fibrosis, Myocardial Infarction, and Heart Failure

Cardiac fibrosis, an element consistent with numerous cardiac conditions, is described as an accumulation of extracellular matrix within the myocardium. Following traumatic instances of cardiomyocyte death, such as myocardial infarction (MI), these myocardial cells are replaced with collagen-based scar tissue [40]. Deposits of collagen are typically identified within the myocardium and the perivascular; over time, this accumulation of collagen can lead to MI and heart failure [40]. There is evidence that increased circulation of pTMAO can lead to a buildup of protein deposit and ultimately fibrosis in the myocardium [41]. Individuals with a history of heart failure present with a significantly higher pTMAO concentration and are associated with increased mortality risk compared to healthy control counterparts [42].

Mice fed a western style diet (WD) have significantly elevated levels of pTMAO [41], diminished cardiac output, and increases in fibrosis [41, 43] relative to their normal diet counterparts. Another study showed four weeks of treadmill exercise rescued losses in cardiac output in mice that underwent a surgically-induced MI [44]. Eight weeks of voluntary exercise while consuming a WD ablated fibrotic increases; however, supplementation of TMAO displayed cardiac parameters similar to their sedentary counterparts [43]. Similarly, sedentary SHRs showed a significantly increased perivascular fibrosis compared to WKY sedentary counterparts. When these SHRs were exercised for 12 weeks, the increased perivascular fibrosis was attenuated; however, SHRs detrained for 4 weeks demonstrated perivascular fibrosis like that of sedentary SHR [28]. Taken together, the current literature demonstrates that increased pTMAO due to dietary intake increases cardiac fibrosis, which can be attenuated with regular exercise as quickly as 8 weeks; however, detraining can revert fibrosis back to baseline levels.

Analyses from mouse, rat, and human gut microbiota post-MI show increased abundances of the families *Eubacteriaceae* and *Lachnospiraceae* [45, 46] and the genera *Phenylobacterium * [44], *Roseateles * [44], *Parabacteroides*, *Lactobacillus*, and *Klebsiella * [45, 47]. However, following MI, human subjects presented differing abundances of *Ruminococcaceae* and *Roseburia * [45, 47]. These alterations may be due in part to the type of MI (ST-segment elevation myocardial infarction (STEMI), non-ST segment elevation myocardial infarction (NSTEMI), etc.) [48]. PCoA plots, which present a graph demonstrating similarities and/or differences among samples, showed MI mice (sedentary or exercised) had distinctly separate clustering compared to control or sham mice that were either sedentary or exercised [44]. Correlational analyses demonstrated that *Helicobacter*, *Prevotella* and *Parabacteroides *were associated with diminished cardiac function and *Ureaplasma *was positively associated with normal left ventricular function [44]. Numerous factors may contribute to alterations in the gut microbiota post-MI, including diet, training status, and overall baseline gut microbiota profiles prior to MI.

Individuals presenting with heart failure possess a significantly different clustering of the gut microbiota compared to healthy controls [49, 50]. These heart failure patients showed increased abundances of *Bacteroides *and *Akkermansia *and decreased abundances of *Ruminococcus *compared to controls; the abundance of *Ruminococcus *was positively associated with increased fiber intake and negatively associated with hypertension [49]. Decreased *Ruminococcus *is consistent with Luedde and colleagues [50], who saw a decrease in *Blautia, Collinsella, Erysipelotrichaceae *in a separate study examining the gut microbiota of individuals with heart failure. Further, Kummen and colleagues [51] found that heart failure patients present with a gut microbiota that has decreased butyrate-producing bacteria and a decreased abundance of *Eubacterium hallii*. While similarities are seen, possible reasons for variation could be linked to the progression of heart failure in patients and compounding CVD complications.

Humans and animals presenting with various types of heart disease consistently demonstrate changes in their gut microbiota, however these increases or decreases in abundances may differ between subject type or by disease status. More research is needed to better understand how changes in gut microbiota abundances may influence disease status or progression.

## 3. There have been Several Proposed Mechanisms Associating the Gut Microbiome with the Development of CVD and Its Associated Conditions, Specifically the Ability for Gut-Derived Metabolites to Influence Host Physiology. Here We will Discuss Potential Gut Microbiota Metabolites that may Positively and/or Negatively Influence CVD Risk and Development.

### 3.1 Trimethylamine and Trimethyl-amine N-oxide

Trimethylamine (TMA) is the diet-derived precursor to TMAO; conversion of TMA to TMAO occurs at the level of the liver [52]. TMAO is a diet- or gut-derived metabolite that has been extensively reviewed as a potential target for drug therapies and dietary intervention due to its strong association with the development of CVD [53]. In 2011, Wang and colleagues [54] were the first to identify significant correlations between three metabolites and CVD risk: TMAO, choline, and betaine. These findings led to the discovery of TMAO derivation from dietary phosphocholine (PC) and the intermediate pathway where PC is converted to choline, to TMA, and then to TMAO [54].

There is limited research investigating the relationship between TMAO levels and exercise. When comparing healthy sedentary males to endurance-trained males, there were no significant differences in pTMAO concentrations before or after a 5-day high fat diet (HFD) intervention [55]. Interestingly, healthy young males following the same treatment had increased pTMAO concentrations 1-4 hours postprandial [56]. It is postulated that potential differences between the 2021 study and Boutagy *et al*.’s 2015 study [56] may lie in the different gut microbiota profiles of the subjects [55]. Sedentary mice fed a WD had significantly higher levels of pTMAO when compared to their voluntary wheel running counterparts. However, when these exercised mice fed a WD were supplemented with TMAO, pTMAO increased to the same level as sedentary mice fed a WD [43]. These studies provide inconclusive support for the role exercise may have on influencing pTMAO levels and warrants further investigation.

Circulating plasma choline and pTMAO concentrations are significantly higher in older mice [21, 57] and adults [26, 57] compared to their young, healthy counterparts. When measuring endothelial function, older adults had significantly less flow compared to younger healthy adults. Further, increased pTMAO concentrations were inversely related to healthy endothelial function [26, 57] and directly related to elevated SBP [57]. To better understand the relationship of age, TMAO, and endothelial dysfunction, Brunt and colleagues [57] fed 6 month (young) and 12 month (old) old mice either a control or TMAO-supplemented diet for 6 months or 3 months, respectively. After 5 months of TMAO supplementation, young mice began developing elevated SBP; by the end of 6 months, young mice displayed significantly increased aortic stiffness compared to control mice. Following one month of TMAO supplementation, older mice began developing elevated SBP and by 3 months also displayed significantly increased aortic stiffness [57]. While it is not feasible to treat diseases with antibiotics, the lack of gut microbiota supports the role of bacteria in the generation of gut-derived metabolites that may expedite disease development.

### 3.2 L-carnitine and γ-butyrobetaine

Levocarnitine (L-carnitine) is a choline analog with conflicting function, showing both cardiac benefits as a treatment [58] and as a precursor to the metabolites TMA and TMAO [59]. Evidence proposes the association between red meat consumption and CVD may not be due to saturated fat [60], but instead due to the gut microbiota’s ability to digest foods into various metabolites, including TMAO [61]. To better study the relationship between L-carnitine and TMAO, Koeth and colleagues [61] created an “L-carnitine challenge”, where groups consumed either a labeled L-carnitine (d3-L-carnitine) supplement or dietary L-carnitine along with the d3-L-carnitine supplement. Following detection of both d3-L-carnitine and d3-TMAO post prandially and 24 hours after the challenge, they examined the involvement of the gut microbiota. Five subjects from the same groups were challenged twice more: the second challenge followed one week of oral broad-spectrum antibiotics and the third was several weeks after antibiotic treatment. Following antibiotic treatment, minimal levels of d3-TMAO were detected. However, results from the third challenge showed similar d3-L-carnitine and d3-TMAO levels to the first, indicating that an intact gut microbiota is necessary for the conversion of L-carnitine into TMAO [61]. Although the same size was small (n = 5) for the follow-up studies, d3-L-carnitine and d3-TMAO levels following gut microbiota depletion and repletion support a strong link between gut bacteria and both L-carnitine and TMAO.

Evidence has emerged that the conversion of L-carnitine into TMAO utilizes an intermediate metabolic step involving γ-butyrobetaine (γBB) [62]. When either vegetarians/vegans or omnivores were challenged with d3-L-carnitine, omnivores presented significantly higher levels of d3-TMAO [61, 63] but not γBB [63]. However, one week of antibiotic treatment did not significantly alter plasma γBB [63]. The limited studies provided collectively demonstrate that both dietary groups can convert L-carnitine to γBB, and the increased pTMAO concentrations may be due to a diet-trained gut microbiota. The lack of changes following antibiotic treatment call for further inquiry to the role the gut microbiota may play in γBB conversion and clearance.

### 3.3 Lipopolysaccharide

Lipopolysaccharide (LPS), a bacteria-derived endotoxin, is released from the outer membrane of gram-negative microbes [64] and is associated with metabolic endotoxemia [65]. Analysis of the gut microbiota from hypertensive patients identified the LPS biosynthesis and export metabolic pathways to be increased compared to healthy controls [15, 29]. Following diagnosis of STEMI, patients presented with significantly elevated levels of circulating LPS [66], indicating a possible increase in abundance of gram-negative bacteria. Using the atherosclerosis mouse model (Apoe -/-), 6-week-old female mice were orally gavaged 5 days/week for 10 weeks with either culture medium (vehicle) or live strains of *Bacteroides vulgatus* and *Bacteroides dorei*. Following the 10-week treatment, mice given either of the Bacteroides strains had significantly less plasma LPS and aortic root lesions compared to the vehicle mice [67]. Despite what we know about altered gut microbiome profiles that are associated with atherosclerosis, these data support the hypothesis that certain bacteria may promote a healthy phenotype in those with CVD.

It has been widely debated whether a diet high in fat, particularly saturated fat consumed through red meat, increases the risks and outcomes of CVD [68, 69, 70]. There is strong evidence supporting increased LPS levels with consumption of a HFD [71, 72]. LPS binding protein (LBP) is a protein and measurable marker of inflammation that binds to LPS and elicits an immune response [73]. After two weeks of either sprint interval or moderate-intensity continuous training, prediabetics and type 2 diabetics saw a significant improvement in LBP [65, 73]. Both training modalities saw a significant decrease in *Bacteroidetes* abundance [65], supporting the decrease in LBP as a majority of *Bacteroides *are gram negative [74]. Mice fed a 60% HFD that underwent swim training 60 minutes/day, 5 days/week for 13 weeks displayed significantly decreased serum LPS levels compared to their sedentary counterparts. The main source of fat for this diet is lard, which is high in saturated fat (Research Diets INC., https://researchdiets.com/formulas/d12492) and supports the effects of exercise on attenuating increased LPS levels induced by a high saturated fat diet.

Much research related to LPS levels and exercise focuses on insulin resistance and tolerance [75, 76], obesity [77], liver disease [78] and immune system intervention [79]. The provided evidence indirectly relates the benefits of exercise to decreased LPS, however, there is no identified literature directly investigating the effects of LPS and exercise in relation to CVD.

### 3.4 Dietary Modifications and Gut-Derived Metabolites

Human dietary classifications include, but are not limited to: (1) vegetarians, which avoid the consumption of all animal flesh (poultry, beef, fish, etc.), (2) vegans, which abstain from the consumption of any animal-derived substance, and (3) omnivores, which consume various animal and plant substances for food [80]. When profiling gut microbiota enterotypes, select omnivores had a higher association with increased pTMAO and a *Prevotella*-enterotype, whereas both vegetarian/vegans were associated with a *Bacteroides*-enterotype and reduced pTMAO concentrations [61]. Omnivorous diets showed significant enrichment of *Peptostreptococcaceae*, *Clostridiaceae*, whereas vegetarian/vegan diets showed significant enrichment of *Lachnispira* [61]. As referenced above, there is a suggestion that individuals following a specific dietary lifestyle may have a diet-trained gut microbiome that can influence, in part, metabolite production.

Mice fed an L-carnitine supplemented diet had a significant and positive correlation with both increased plasma TMA/TMAO concentrations and *Prevotella*, *Deferribacterales*, *Tenericutes* and a negative correlation with decreased pTMAO concentrations and *Bacteroidetes* [61]. Seeking to identify specific bacteria involved in the metabolism of L-carnitine into TMA, Koeth and colleagues [63] created five different “species pools” containing multiple bacterial strains. The combination of *Eggerthella lenta *with one of the following (*Hungatella hathewayi*, *Bacteroides dorei*, *Emergencia timonensis*, *Peptoniphilus indolicus*) were found to be specifically required for the conversion of L-carnitine to TMA. The identification of specific bacterial species’ interactions in gut microbiome-specific metabolic pathways may be the future of individualized medicine, however there is much more research to be done in this area.

**Fig. 1.**
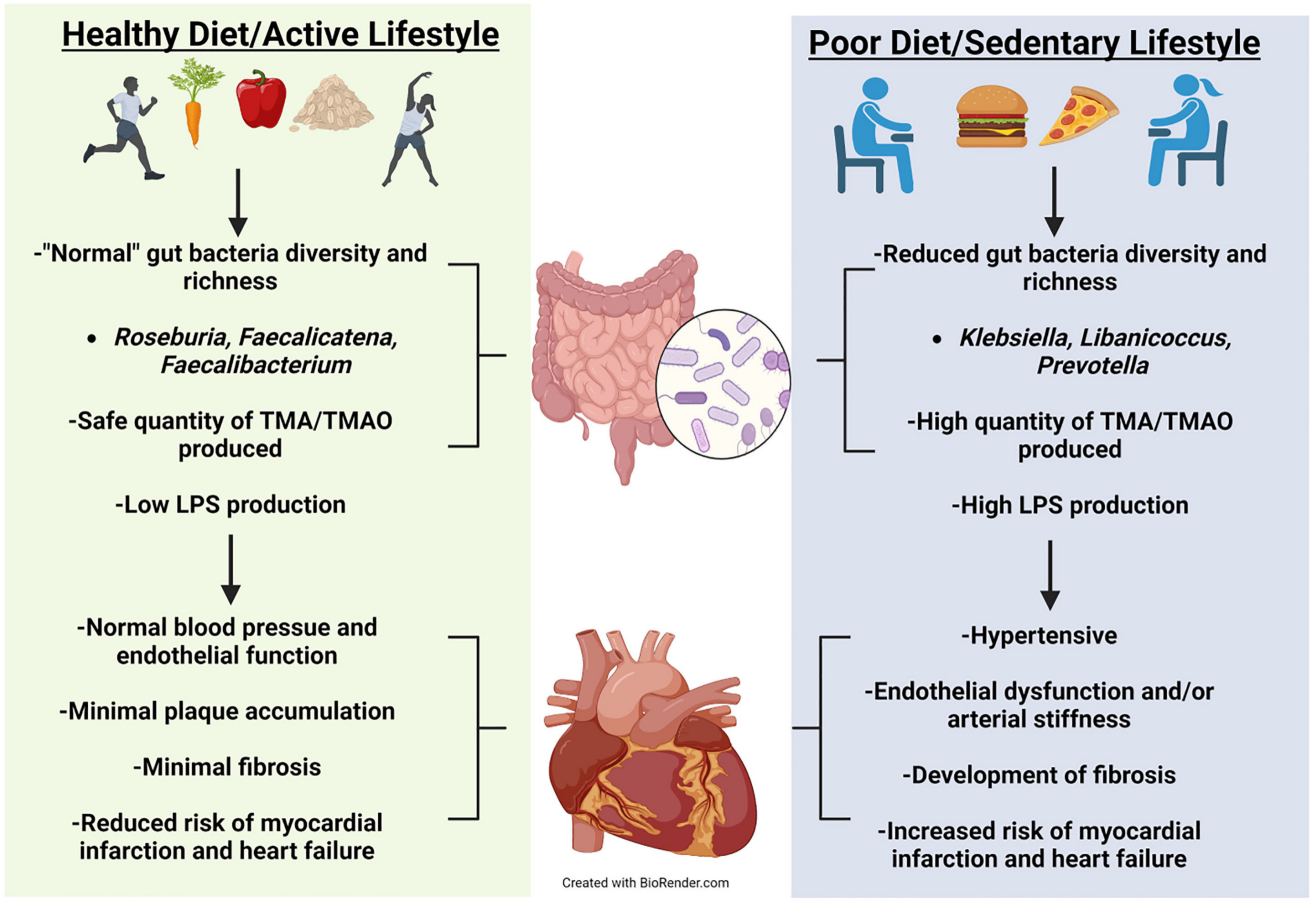
**Conceptual Model**. Healthy diet and exercise are well known to manifest a beneficial microbiota that can optimize health. Positive cardiovascular outcomes have been correlated to a healthy diet and exercise. Evidence has emerged showing that healthy diet and exercise produce beneficial gut microbes that have reduced production of harmful metabolites manifesting normal cardiovascular function. Alternatively, poor diet and sedentary behavior can increase pathogenetic microbes leading to unfavorable cardiovascular outcomes.

Apoe -/- mice fed an L-carnitine-supplemented diet for 15 weeks possessed almost double the aortic root atherosclerotic plaque compared to their control diet counterparts [61]. Further, Apoe -/- mice fed the same L-carnitine supplemented diet with antibiotics had significantly less atherosclerotic plaque compared to their gut intact counterparts [61]. This demonstrates that the gut microbiota is likely involved in plaque development when L-carnitine is high. The Apoe -/- model was utilized by Wang and colleagues [54] to observe atherosclerotic plaque development in mice on a normal diet, an intermediate choline (0.5%) supplemented diet, a high choline (1.0%) supplemented diet, or a TMAO (0.12%) supplemented diet. When the mice reached 20 weeks old, all three supplemented diet groups had significantly increased amounts of atherosclerotic plaque buildup and pTMAO levels [54], demonstrating that there may be more than one possible source when examining specific causes of disease. Using deuterium labeled (d9)-TMAO, d9-phosphocholine (d9-PC), and d9-choline, mice pretreated for 3 weeks with antibiotics did not present with plasma d9-TMAO when orally gavaged with either d9-PC or d9-choline. However, mice orally gavaged with d9-PC possessing an intact gut microbiota did [54]. When antibiotic-treated mice were conventionalized with normal mice for 4 weeks and retested with the same oral gavage of either d9-PC or d9-choline, d9-TMAO was able to be measured in both treatments [54]. Both male and female Apoe -/- mice fed a 1% choline supplemented diet and treated with antibiotics possessed significantly less aortic lesions at 20 weeks old compared to their gut-intact counterparts [54]. The absence of the gut microbiota and reduction of pTMAO, atherosclerotic plaque, and aortic lesions further supports the involvement of gut bacteria in not only host physiological health but also the derivation of intermediate and endpoint metabolites. How these metabolites may positively or negatively influence CVD requires further investigation, see Conceptual Model (Fig. 1).

## 4. Various Interventions have been Proposed to Reduce CVD and Its Associated Risk Factors. Here, We will Discuss These Interventions and Their Respective Supporting Evidence. 

### 4.1 Lifestyle Modifications-Diet and Physical Activity

The benefits of exercise on various types of CVD have been extensively reviewed [81, 82, 83, 84], however, the influence of the gut microbiota on both is limited [85]. Sedentary behavior is a modifiable risk factor for CVD and therefore can be altered through behavior modification. CVD is closely associated with the accompanying risk factors of obesity and T2D [5, 6]. Obese individuals consuming a hypocaloric diet alongside a 5 day/week exercise protocol had a greater reduction in pTMAO relative to those following the same exercise routine but maintaining their typical caloric intake [86]. A prospective study examining adults with T2D found an inverse relationship of TMAO levels and physical activity status [87]. Significant decreases in systolic and diastolic blood pressure (DBP), but not pTMAO, can be seen within one week through lifestyle interventions including: a plant-based diet, aerobic exercise, and stress and nutrition management classes [88].

WDs are typically low in fiber and have been long associated with increased incidences of chronic disease development and an increased risk for CVD [89, 90]. Further, heart failure patients tend to have significantly lower fiber intake relative to controls [49]. Mice exercised while consuming a WD for 8 weeks had significantly lower body weights compared to their sedentary counterparts, even when supplemented with TMAO; however, exercise did not attenuate high pTMAO concentrations in the supplemented WD [43]. Women consuming a Paleolithic diet, which excludes cereals, legumes, and dairy, for 4 weeks, did not affect serum TMAO levels compared to individuals following the Australian Guide to Healthy Eating [91]. Long-term (over one year) Strict Paleolithic diet individuals possessed significantly higher levels of TMAO, significantly lower abundances of *Bifidobacterium *and *Roseburia*, and significantly higher abundances of *Hungatella *compared to healthy controls [92]. These data support that changes to TMAO may require a longer period of dietary intervention to significantly change host levels. Despite exercise’s ability to improvement cardiovascular health, more work is needed in this area to determine if exercise-mediated changes to the gut microbiota drive positive cardiovascular outcomes. Further, it is unclear if exercise has the capacity to lower pTMAO levels if individuals have poor dietary habits.

### 4.2 Fecal Microbiota Transplant

The use of fecal microbiota transplants (FMTs) as a treatment and therapeutic has been extensively reviewed [93, 94, 95, 96]. Fecal samples from three human patients (two hypertensive and one healthy) were orally inoculated into germ free mice. Mice inoculated with hypertensive fecal samples displayed significantly elevated SBP and enrichment of *Prevotella* and *Coprobacillus* and decreased levels of *Coprococcus* and *Roseburia *(identified in hypertensive human patients) compared to the control fecal sample recipient [15]. These data support the hypothesis that the gut microbiota has a distinct profile in hypertensive patients and may influence disease phenotype.

One-week post-FMT from donor WKY rats (control) to recipient SHRs and from donor SHRs to recipient WKY rats showed a significant decrease and a significant increase in SBP, respectively [97, 98]. FMT from donor SHRs that had been exercised or exercised and detrained for 4 weeks into normal SHRs showed a significantly decreased SBP compared to sedentary SHRs [28]. FMT donor sedentary SHRs showed increased perivascular fibrosis that was not seen in the FMT from exercised SHR [28]. This demonstrates that a trained (or untrained) gut microbiota can influence SBP and perivascular fibrosis development.

## 4.3 Prebiotics

Prebiotics are broadly defined as non-digestible foods that provide host benefits through changes to the intestinal flora [99]. While there are several types of prebiotics, many fall into the following categories: oligosaccharides (including galacto-oligosaccharide), fructans (including inulin and fructo-oligosaccharides [FOSs]), fiber, and starch [100]. The beneficial effects of prebiotics on gut microbiota composition have been well-reviewed [101, 102, 103, 104]. In particular, prebiotics improve the production of gut-derived metabolites such as SCFAs [105] and increase the abundance of beneficial bacteria such as butyrate-producing *F. prausnitzii* [106, 107], *Akkermansia muciniphila *(*A. muciniphila*) [106, 107], and Bifidobacteria [108, 109]. Prebiotics have been indirectly associated with decreases in CVD risk and development by attenuating inflammatory, gut-derived compounds like LPS, reducing chronic inflammation, or controlling for comorbidities such as obesity and T2D [110, 111, 112], all of which are associated with improved gut health.

Fructans are polymers of fructose linked by β-2,1 bonds; inulin is characterized as having longer polymerized chains, whereas FOS possesses shorter polymerized chains [35]. Rats fed a HFD for 12 weeks showed increased cardiomyocyte hypertrophy compared to rats on a control diet; however, this hypertrophy was not reversed with supplementation of FOS alongside the HFD [113]. CVD is an accumulation of long-term lifestyle habits; it is possible that 12 weeks of FOS was not a long enough intervention to see significant changes in cardiomyocyte physiology. One clinical trial protocol aims to elucidate the potential benefits of inulin in adults at risk of T2D and associated cardiometabolic health. This publication provides a rationale and design for the study; however, there is no follow up or subsequent data since the original release in 2015 [114]. Ultimately, long-term research needs to be conducted in both animals and humans to better understand the effects of prebiotic fructans on CVD risk and development.

Resistant starch (RS) is classified as a prebiotic due to its inability to be digested in the upper gut [100]. Human subjects fed a maize-derived whole grain cereal saw significant increases in abundances of Bifidobacteria after 21 days of intervention compared to their respective baseline abundances [109]. Increased Bifidobacteria abundances have been associated with improving disease states of cancer and production of metabolites such as SCFAs and antimicrobial peptides [108], which may indirectly decrease risk of CVD.

Dietary fiber is an edible carbohydrate polymer and can be classified as either insoluble, forgoing degradation during digestion, or soluble [115]. The benefits of dietary fiber on CVD risks and outcomes from observational studies has been well-reviewed [116, 117, 118, 119, 120]. Many of these reviews support positive associations between increased dietary fiber consumption and decreased CVD risk. However, there is belief that these benefits may come from multiple factors such as other nutrients within the foods, improved metabolic measures such as lipid profiles, and increased abundances of beneficial gut bacteria. Male Apoe -/- mice were fed a HFD for 8 weeks to induce atherosclerotic plaques and endothelial dysfunction in the aorta; following the 8-week feeding period, mice were then divided into either HFD, HFD + 5% chitin-glucan (an insoluble fiber), or HFD + 5% chitin-glucan and 0.5% polyphenol-rich pomegranate peel extract (PPE). Mice provided a HFD + 5% chitin-glucan and 0.5% PPE displayed significantly increased levels of heme-nitrosylated hemoglobin in the blood and western blot analyses confirmed endothelial nitric oxide synthase was significantly increased in the mesenteric arteries compared to the HFD or HFD + 5% chitin-glucan groups; both are markers of improved endothelial function. The HFD increased abundances of both *Alistipes *and *Lactobacillus *species in the cecum, which was rescued by supplementing the HFD with both chitin-glucan and PPE. Further, *A. muciniphila *was significantly decreased in the HFD + 5% chitin-glucan and 0.5% PPE when compared to the HFD alone [121]. Authors were unsure why *A. muciniphila* decreased; however, there is evidence that mice fed a HFD and consuming fiber have decreased abundances of *Akkermansia* [122]. Differences may also be attributed to fiber type and the primary source of fat supplementation in the modified diet. The combination of insoluble fiber and PPE support the hypothesis that it is a well-rounded diet, not necessarily a single compound, that has the biggest contribution to host health and an improved gut microbiota profile [123, 124].

Marques and colleagues [125] utilized a hypertensive mouse model to investigate the direct link of fiber to the gut microbiota and development of hypertension and heart failure. When compared to their hypertensive control counterparts, fiber-fed hypertensive mice had significantly decreased SBP, DBP, mean arterial pressure (MAP), and heart: body weight ratios, which was due to attenuation of cardiac hypertrophy. Further, when compared to their hypertensive control counterparts, fiber-fed hypertensive mice showed significantly decreased diastolic left ventricular internal dimension and fractional shortening, both measurements of cardiac function, and significantly decreased cardiac fibrosis. Lastly, fiber-fed hypertensive mice displayed a unique gut microbiota profile compared to their counterparts; in particular, there was a significant increase in acetate-producing bacteria [125]. Acetate is the most abundant SCFA found in the human body; increased acetate can contribute to increased production of butyrate, a significant contributor to colonocyte energy and gut health [126]. While this study appears to provide succinct evidence of a beneficial prebiotic-gut microbiota-CVD interaction, there is a lack of exercise inclusion. There appears to be no research attempting to directly link prebiotic intake to CVD risk, exercise, and the gut microbiota, despite the known benefits of exercise.

### 4.4 Probiotics

The Food and Agriculture Organization of the United Nations and the World Health Organization define a probiotic as ‘live microorganisms which when administered in adequate amounts confer a health benefit on the host’ [127]. Probiotics can contain a single strain or multiple strains and normally belong to one of the following genera: *Streptococcus*, *Enterococcus*, *Lactococcus*, *Lactobacillus*, *Bacillus*, *Bifidobacterium*, and the yeast genus *Saccharomyces * [128]. Zhou and colleagues [129] nicely review probiotic-supporting literature related to reduced CVD or decreased CVD risk factors up to 2020. Patients with T2D provided a combination of *Lactobacillus acidophilus*, *Lactobacillus fermentum*, *Lactobacillus gasseri*, *Lactiplantibacillus plantarum *(*L. plantarum*), and a prebiotic showed significant decreases in MAP [130]. We are curious if decreased MAP would occur in subjects only provided the probiotic without fiber supplementation, as addition of fiber in hypertensive mice also displayed reduced MAP [125]. Female mice fed a 1% choline diet and orally gavaged with a multi-strain formula of Lactic Acid Bacteria (LAB) including *L. plantarum*, *Limosilactobacillus fermentum *(*L. fermentum*), and *Lactobacillus amylovorus *(*L. amylovorus*) displayed significantly decreased levels of serum TMA and TMAO after 7, 14, and 28 days compared to mice fed only a 1% choline diet. When investigated independently in mice consuming a 1% choline diet, *L. plantarum* significantly decreased serum TMA and TMAO levels after 14 and 21 days compared to mice fed only a 1% choline diet. Further, *L. amylovorus *significantly decreased serum TMAO after 7 days and both TMA and TMAO after 14 days; *L. fermentum *significantly decreased serum TMA levels after 14 days [131]. LAB are commonly found in fermented foods such as vegetables, meat, dairy, and cereals and have been touted to provide numerous health benefits [132]. One meta-analysis of 10 studies found that fermented dairy foods, particularly cheese and yogurt, displayed decreased CVD risk [133]. It is interesting that the three different LAB did not decrease serum TMA and TMAO levels at the same rate independently and supports the hypothesis that bacteria within the gut work best in functional communities [16]. Human fecal samples from control, coronary artery disease, and heart failure subjects analyzed for LPS concentrations found that LPS from *Bacteroides *was structurally different and significantly lower, and promoted less pro-inflammatory cytokines when compared to *E. coli *LPS [134]. While *Bacteroides *is not typically considered a ‘good’ bacteria [74], the authors make a strong case for more research to investigate individual characteristics and roles bacteria may play.

Gut distress and discomfort are common problems reported in endurance athletes and have been attributed to disruption in tight junction proteins, ischemia, and dietary intake prior to exercise. Exercise has also been shown to improve gut microbiota diversity, particularly SCFA-producing bacteria, and increase production of SCFAs [3, 4]. Male mice given *L. plantarum *for six weeks in low or high doses saw significantly improved grip strength via a forelimb force transducer and results in a swim to exhaustion test; mice provided a highest dose of *L. plantarum *significantly outperformed both their low dose and vehicle counterparts. Probiotic-supplemented mice also had a significant and dose-dependent increase in the number of type I muscle fibers in the gastrocnemius compared to their vehicle counterparts, with mice provided the highest dose of *L. plantarum *displaying the most type I fibers [135]. Experienced triathletes provided *L. plantarum *showed significant improvement in maximal oxygen uptake and significantly increased levels of acetic, propionic, and butyric acid compared to their placebo counterparts [136]. Healthy normal human subjects given either a low or high dose of *L. plantarum *for 6 weeks saw significant improvements in time to exhaustion compared to the placebo group, with those receiving the high dose significantly outperforming their low dose counterparts. Further, high dose subjects also saw a significant decrease in body fat mass and significant increase in muscle mass [137]. *L. plantarum *has been well-studied and findings provide strong support for its role in improved gastrointestinal disorders and production of protective antimicrobial compounds [138]. Much of the research investigating the benefits of probiotics in athletes focus on prevention of illness and inflammation [139, 140]. Ultimately, the International Society of Sports Nutrition acknowledge the potential benefits of probiotics in the exercising and athletic population but request more research in this field [141].

It is not uncommon for probiotic companies to reach out to laboratories for experimental assistance and expertise; however, it should be noted that these collaborations may insinuate possible conflicts of interest within studies [142]. Healthy young males on 4-week, HFD hypercaloric diet supplemented with the probiotic VSL#3 did not see an improvement in pTMAO levels compared to their placebo counterparts [143]. VSL#3 (https://www.vsl3.com/) is designed for individuals with irritable bowel syndrome and ulcerative colitis and therefore may not contain bacteria that confer benefits for CVD. It is important to properly foster these relationships between industry and academia to provide adequate research funding and reliable results, particularly in a field where probiotics are minimally regulated [144].

## 5. Conclusions

As the gut microbiota continues to link to itself to CVD, it is paramount to investigate these relationships at various stages: the pre-diseased, the diagnoses, and in the continued follow ups. Predictable gut microbiota profiles are in their early stages of disease diagnosis; however, evidence has found strong correlations between current and potential disease outcomes and gut bacteria present [13, 14, 15]. Additional work examining how associated gut-derived metabolites, such as carnitine, TMA/TMAO, and LPS may contribute to disease development will shed light on the mechanisms in which gut bacteria utilize dietary resources to potentially facilitate CVD. From the opposite approach, studies investigating how prebiotics may contribute to a healthy gut microbiota and therefore a healthy host, possibly through SCFA production, will compliment CVD research. Probiotics and FMT are in their early stages of investigation when relating to CVD; additional research will expand on the application of these potential treatments as a means to manipulate the gut microbiota into a more conducive profile for improved host health. Unfortunately, the bacteria identified in the gut microbiota of animals does not always mirror the human gut microbiota and must be accounted for as treatments are developed.

Despite the numerous contributors to CVD development listed in this review, lifestyle changes such as diet modification and exercise in various modalities and intensities appears to be a strong deterrent of CVD severity and a key component of prevention. Modifications in physical activity alone have consistently shown improvements in blood pressure and improved gut microbiota diversity in both animal models and humans, as mentioned in this review. Further, exercise in combination with caloric and macronutrient adjustment have the capacity to alter prominent gut microbiota metabolites such as TMAO [86]; future studies will hopefully explore how exercise may change other CVD-associated metabolites. While it is rather simple to collect a fecal sample, measure food consumed, and calculate exercise intensity in animals, humans are much more complex. The animal work completed thus far has been invaluable; however, human studies will provide the best evidence of how CVD development and outcome may be linked to the gut microbiota and how these can be improved or worsened following lifestyle interventions.

Evidence supports those individuals consuming whole foods present with reduced instances of CVD and associated diseases, increased abundances of beneficial gut bacteria, and altered metabolite production, particularly those known to be gut-derived [99, 104, 123, 124]. Further, exercise in varying modalities and general lifestyle changes to increase physical activity demonstrate improved health outcomes related to CVD risk and comorbidities and microbial diversity [3, 4, 83, 84]. It is difficult to study an outcome such as CVD when including multiple factors (exercise, the gut microbiota, gut microbiota-influencing foods); however, it is evident that these variables contribute to improved health in their own ways. Therefore, future studies should include analyses of how these important factors can not only be studied in unison, but how they can eventually be incorporated into a personalized healthy lifestyle based on individual needs.
